# Human Pituitary Organoids: Transcriptional Landscape Deciphered by scRNA‐Seq and Stereo‐Seq, with Insights into SOX3's Role in Pituitary Development

**DOI:** 10.1002/advs.202414230

**Published:** 2025-02-14

**Authors:** Shengjie Wang, Deyue Jiang, Yan Xiao, Qiaozhen Qin, Heyang Zhang, Lingtong Ye, Jide Jin, Xiaoxia Jiang, Qinghua Guo

**Affiliations:** ^1^ Department of Endocrinology the First Medical Center of Chinese PLA General Hospital Beijing 100853 China; ^2^ Beijing Institute of Basic Medical Sciences 27 Taiping Road of Haidian District Beijing 100850 China; ^3^ Beijing Institute of Radiation Medicine 27 Taiping Road of Haidian District Beijing 100850 China

**Keywords:** organoids, pituitary, scRNA‐seq, SOX3, stereo‐seq

## Abstract

The 3D human pituitary organoid represents a promising laboratory model for investigating human pituitary diseases. Nonetheless, this technology is still in its nascent stage, with uncertainties regarding the cellular composition, intercellular interactions, and spatial distribution of the human pituitary organoids. To address these gaps, the culture conditions are systematically adjusted and the efficiency of induced pluripotent stem cells’ (iPSCs’) differentiation into pituitary organoids is successfully improved, achieving results comparable to or exceeding those of previous studies. Additionally, single‐cell RNA‐sequencing (scRNA‐seq) and stereomics sequencing (Stereo‐seq) are performed on the pituitary organoids for the first time, and unveil the diverse cell clusters, intricate intercellular interactions, and spatial information within the organoids. Furthermore, the SOX3 gene interference impedes the iPSCs’ differentiation into pituitary organoids, thereby highlighting the potential of pituitary organoids as an ideal experimental model. Altogether, the research provides an optimized protocol for the human pituitary organoid culture and a valuable transcriptomic dataset for future explorations, laying the foundation for subsequent research in the field of pituitary organoids or pituitary diseases.

## Graphical abstract

An optimized protocol is developed to differentiate human iPSCs into pituitary organoids and the cellular composition, intercellular interactions, and spatial organization within pituitary organoids are characterized via scRNA‐seq and Stereo‐seq. SOX3 interference impaires human iPSCs’ differentiation into pituitary organoids, highlighting the potential of pituitary organoids as an ideal laboratory model for pituitary disease research.

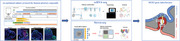

## 1 Introduction

The pituitary gland is one of the most crucial endocrine organs in the human body, regulating a wide range of physiological processes, including stress response, homeostasis, metabolic regulation, growth, development, and reproduction.^[^ ^1^, ^2^, ^3^ ^]^ However, pituitary function can be disrupted by various factors, such as tumors, trauma, radiotherapy, infection, drugs, and genetic anomalies.^[^ ^4^, ^5^, ^6^, ^7^ ^]^ The gland's unique anatomical position, diminutive size, and the ethical challenges associated with procuring human tissue samples pose significant obstacles to in‐depth study of the pituitary. Previous studies have relied on pituitary cell lines or animal models, but these models often fail to accurately reflect the human pituitary, underscoring the pressing need for a more suitable and representative laboratory model to investigate human pituitary diseases effectively.

In recent years, the development of organoids has significantly advanced the fields of disease modeling, drug screening, and regenerative medicine by enabling the creation of miniature organs or tissues through 3D cell culture in vitro.^[^ ^8^, ^9^, ^10^, ^11^ ^]^ Pituitary organoids, in particular, offer substantial potential as a research tool,^[^ ^10^, ^12^ ^]^ because they can circumvent species‐specific differences observed in animal models and more effectively simulate the intricate cellular interactions absent in traditional 2D cultures.^[^ ^13^ ^]^ Despite this promise, the development of pituitary organoid culture protocols has been hampered by technical challenges and experimental complexities. Early attempts to differentiate pluripotent stem cells into 3D pituitary organoids yielded limited success, as evidenced by the sparse presence of adrenocorticotropic hormone (ACTH)‐positive cells in the organoids.^[^ ^14^ ^]^ This implies that the methodologies for generating pituitary organoids remain in their early stages, characterized by low differentiation efficiency, and necessitate further refinement. Therefore, the first objective of our study is to address this gap by developing a mature and stable protocol for pituitary organoid culture.

The limited understanding regarding the cellular composition and spatial organization of pituitary organoids has considerably hindered their application in research. In this regard, single‐cell RNA sequencing (scRNA‐seq) and stereomics sequencing (Stereo‐seq) have emerged as indispensable instruments in contemporary biomedical research,^[^ ^15^, ^16^ ^]^ facilitating a comprehensive comprehension of distinct cell types and their spatial arrangement within tissues and organs. Deciphering the transcriptional landscape of pituitary organoids using scRNA‐seq and Stereo‐seq may offer valuable insights into the cellular composition and spatial organization of pituitary organoids. Therefore, the second objective of our study is to fill this gap by conducting scRNA‐seq and Stereo‐seq analyses on pituitary organoids, laying a foundation for further research and advancing their applications in pathogenesis studies, regenerative medicine, and drug development.^[^ ^17^ ^]^

The development of pituitary gland is regulated both temporally and spatially by a series of specific transcription factors and signal transduction pathways.^[^ ^1^ ^]^ SOX3 (SRY‐box transcription factor 3) is a crucial and indispensable transcription factor in the formation of the hypothalamic‐pituitary axis^[^ ^18^ ^]^ and serves as a dose‐dependent regulator for SHH (sonic hedgehog) transcription.^[^ ^19^ ^]^ Carreno et al. demonstrated that *Shh* in the hypothalamus was crucial for the fate determination and proliferation of *Lhx3* (LIM homeobox 3)‐positive pituitary progenitor cells.^[^ ^20^ ^]^ Defects in the SOX3 gene have been associated with congenital pituitary hypoplasia and combined pituitary hormone deficiency (CPHD).^[^ ^21^, ^22^, ^23^ ^]^ These studies predominantly utilized the gene knockout mouse models for research, suggesting that SOX3 might play an important role in pituitary development through the SHH/LHX3 signaling pathway. However, notable interspecies differences exist between murine and human pituitaries. Thus, in this study, our third objective is to investigate the impacts of SOX3 gene interference on pituitary development using the human pituitary organoid model, with the goal of validating the utility of pituitary organoids as a suitable experimental model for studying human pituitary diseases.

In summary, our research is centered on three primary objectives: optimizing the culture protocol of human pituitary organoids, elucidating their cellular composition and spatial arrangement through scRNA‐seq and Stereo‐seq, and investigating the impacts of SOX3 gene interference on the development of pituitary organoids.

## 2 Results

### 2.1 Optimizing the Culture Protocol of 3D Pituitary‐Placode Organoids

The pituitary placode, originating from the non‐neuroectoderm, undergoes a sequential expression of transcription factors PITX1 (Paired Like Homeodomain 1) and LHX3 during early and late stages, respectively, and subsequently differentiates into the anterior pituitary.^[^ ^24^, ^25^, ^26^, ^27^ ^]^ Given the critical role of the pituitary placode in pituitary gland formation, the successful generation of pituitary organoids from pluripotent stem cells relies on the successful induction of pituitary placode organoids.

**Figure 1:**
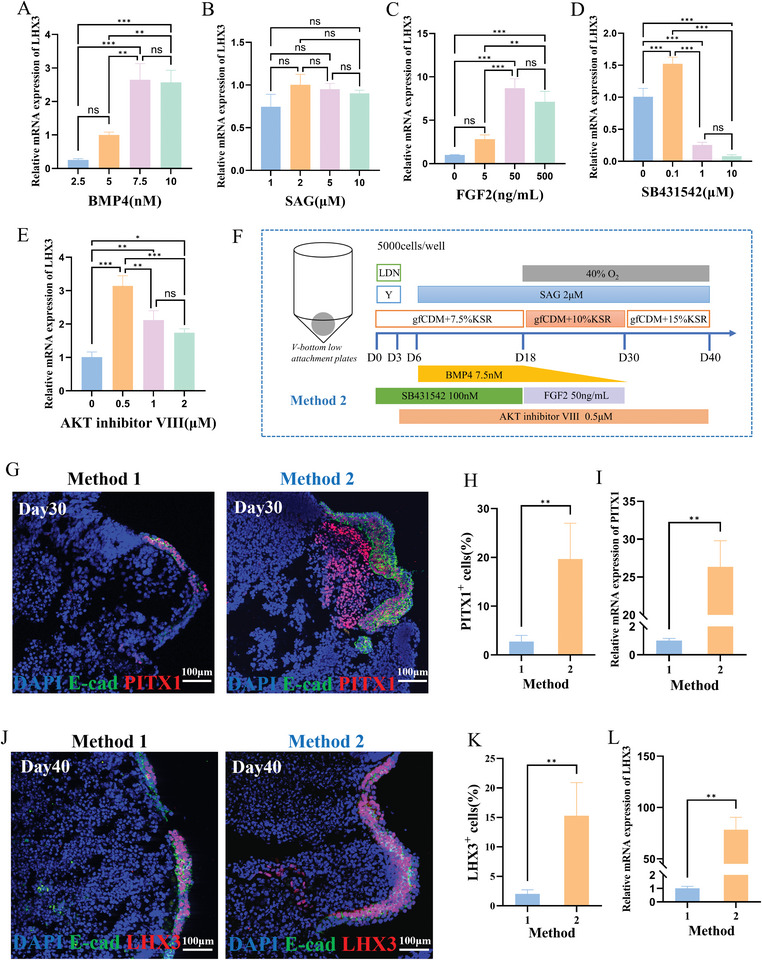
Optimization of the culture protocol for 3D pituitary‐placode organoids. A‐E) Impact of altering the concentrations of BMP4 (A, day6‐18), SAG (B, day6‐40), FGF2 (C, day18‐30), SB431542 (D, day0‐18), and AKT inhibitor VIII (E, day3‐40) based on method 1 on the relative expression levels of LHX3 mRNA in organoids on day 40 (N = 3, mean ± SD, one‐way ANOVA test with post hoc Bonferroni's method). F) Optimized culture protocol for 3D pituitary‐placode organoids, named as the Method 2. G) PITX1 expression of organoids cultured by methods 1 and 2 on day 30, revealed by immunofluorescence staining (E‐cad: E‐cadherin; Scale bars: 100 µm). H) percentage of PITX1‐positive cells (N = 5, mean ± SD, Two‐tailed Welch's t‐test) and (I) PITX1 mRNA expression (N = 3, mean ± SD, Two‐tailed Welch's t‐test) within organoids cultured by methods 1 and 2 on day 30. J) LHX3 expression of organoids cultured by methods 1 and 2 on day 40, revealed by immunofluorescence staining (Scale bars: 100 µm). K) percentage of LHX3‐positive cells (N = 5, mean ± SD, Two‐tailed Welch's t‐test) and (L) LHX3 mRNA expression (N = 3, mean ± SD, Two‐tailed Welch's t‐test) within organoids cultured by methods 1 and 2 on day40. ns means no significance, ^*^ *p* < 0.05, ^**^ *p* < 0.01, and ^***^ *p* < 0.001.

Based on the previously reported protocol for differentiating human induced pluripotent stem cells into pituitary placode organoids,^[^ ^28^, ^29^ ^]^ we initiated our investigation with a slightly adjusted concentration of KnockOut Serum Replacement (KSR), termed Method1 (Figure S1A, Supporting Information). However, the organoids obtained using Method1 exhibited suboptimal differentiation, as indicated by the low expression levels of PITX1 and LHX3. Considering the important roles of Bone Morphogenetic Protein (BMP) signaling pathway and SHH signaling pathway in the development of the pituitary gland, we first investigated whether the addition of BMP4 and SAG (Smoothened Agonist) is necessary for the differentiation of iPSCs into pituitary placode organoids. The results showed that the addition of BMP4 from days 6 to 18 and SAG from days 6 to 40 significantly enhanced the expression of LHX3 mRNA in the organoids on day 40 (Figure S1B, Supporting Information). Next, we further explored the optimal concentrations of BMP4 and SAG. Based on Method1, we systematically modified the concentrations of BMP4 from day 6 to 18 (**Figure** **1**A) and SAG from day 6 to 40 (Figure 1B), respectively. By assessing the expression levels of LHX3 mRNA in the organoids on day 40, we determined the optimal concentrations of BMP4 and SAG to be 7.5 nM and 2 µM, respectively.

Furthermore, several intervention factors could be utilized to enhance the efficacy of the culture protocol. Timely withdrawal of Noggin in the dSMAD‐inhibition protocol to relieve the inhibition of the BMP signaling pathway promoted the differentiation of pluripotent stem cells into the SIX1^+^ pre‐placode ectoderm.^[^ ^30^ ^]^ The activation of the fibroblast growth factor (FGF) signaling pathway facilitated the development of the pituitary,^[^ ^31^ ^]^ while AKT signaling inhibitors promoted the differentiation of pluripotent stem cells into the hypothalamic lineages by antagonizing the insulin‐induced activation of the PI3K/AKT signaling pathway.^[^ ^32^ ^]^ Therefore, we explored the effects of introducing FGF2, SB431542 (TGF‐β signaling inhibitor), and AKT inhibitor VIII to enhance the differentiation of iPSCs into pituitary‐placode organoids. Based on Method1, we adjusted the concentrations of FGF2 from day 18 to 30 (Figure 1C), SB431542 from day 0 to 18 (Figure 1D), and AKT inhibitor VIII from day 3 to 40 (Figure 1E). By evaluating the expression levels of LHX3 mRNA in the organoids on day 40, we determined the optimal concentrations of FGF2, SB431542, and AKT inhibitor VIII to be 50ng mL^−1^, 0.1 µM, and 0.5 µM, respectively. Consequently, we refined our approach and established an optimized pituitary‐placode organoid culture protocol, denoted as Method2 (Figure 1F).

To validate the efficacy of Method2, organoid samples obtained on day 30 and day 40 using Methods 1 and 2 were collected. Immunofluorescence staining was employed to compare the percentage of PITX1‐positive cells on day 30 and LHX3‐positive cells on day 40 between Methods 1 and 2 (Figure 1G,J). Additionally, quantitative PCR experiments were conducted to assess the relative expression levels of PITX1 mRNA on day 30 and LHX3 mRNA on day 40 for Methods 1 and 2 (Figure 1I,L). Our findings revealed that organoids cultured using the improved Method2 exhibited a significantly higher percentage of PITX1‐positive cells and relative expression levels of PITX1 mRNA on day 30, as well as a higher percentage of LHX3‐positive cells and relative expression levels of LHX3 mRNA on day 40, compared to those cultured using Method1 (Figure 1G–L). This underscores the effectiveness of systematically optimizing the culture conditions, resulting in a substantially enhanced efficiency of iPSCs’ differentiation into pituitary‐placode organoids.

### 2.2 Comparison between Initial and Improved Culture Protocols for Pituitary Organoids

**Figure 2:**
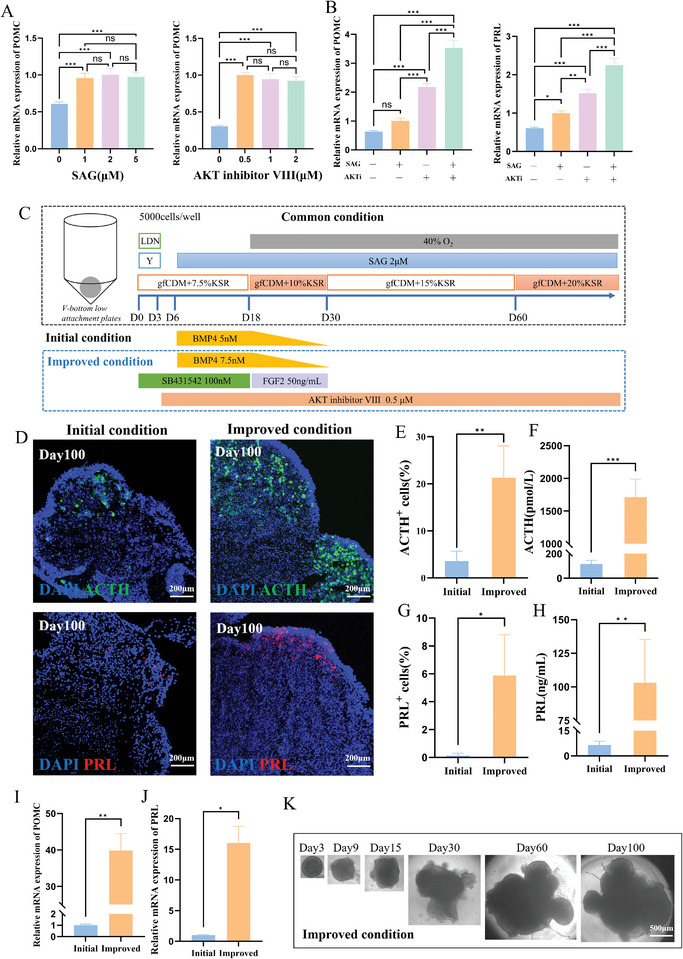
Comparison between the initial and improved culture protocols for pituitary organoids. A) Impact of adjusting the concentrations of SAG or AKT inhibitor VIII after day40 on the relative expression levels of POMC mRNA within organoids on day100 (N = 3, mean ± SD, one‐way ANOVA test with post hoc Bonferroni's method). B) Impact of adding 2 µM SAG and/or 0.5 µM AKT inhibitor VIII after day40 on the expression levels of POMC and PRL mRNA within organoids on day 100 (N = 3, mean ± SD, one‐way ANOVA test with post hoc Bonferroni's method). C) Details about the initial and improved culture protocols for pituitary organoids. D) ACTH and PRL expression of organoids cultured by the initial and improved protocols on day 100, revealed by immunofluorescence staining (Scale bars: 200 µm). E) Percentage of ACTH‐positive cells (N = 5, mean ± SD, Two‐tailed Welch's t‐test) and (F) ACTH secretion levels (N = 5, mean ± SD, Two‐tailed Welch's t‐test) within organoids cultured by the initial and improved protocols on day 100. G) Percentage of PRL‐positive cells (N = 5, mean ± SD, Two‐tailed Welch's t‐test) and (H) PRL secretion levels (N = 5, mean ± SD, Two‐tailed Welch's t‐test) within organoids cultured by the initial and improved protocols on day 100. I) POMC and J) PRL mRNA expression of organoids cultured by the initial and improved protocols on day 100 (N = 3, mean ± SD, Two‐tailed Welch's t‐test). K) Bright‐field images of the organoids cultured by the improved protocol (Scale bars: 500 µm) ns means no significance, ^*^ *p* < 0.05, ^**^ *p* < 0.01, and ^***^ *p* < 0.001.

To explore the necessity of continued supplementation with SAG and AKT inhibitor VIII for the 3D pituitary‐placode organoids cultured using Method2, we adjusted the concentrations of SAG and AKT inhibitor VIII after day 40 (Figure S1C, Supporting Information). By evaluating the expression levels of Pro‐opiomelanocortin (POMC) mRNA in the organoids on day 100, we determined the optimal concentrations of SAG and AKT inhibitor VIII to be 2 µM and 0.5 µM, respectively (**Figure** **2**A). Additionally, we sought to discern whether the concurrent addition of SAG and AKT inhibitor VIII had synergistic or antagonistic effects on organoid induction. After day 40, we divided the organoids cultured using Method 2 into four groups: group 1 without SAG or AKT inhibitor VIII, group 2 with 2 µM SAG, group 3 with 0.5 µM AKT inhibitor VIII, and group 4 with both 2 µM SAG and 0.5 µM AKT inhibitor VIII (Figure 2B). Notably, the relative expression levels of POMC and PRL (Prolactin) mRNA on day 100 were significantly elevated in Group 4 compared to Groups 1 to 3, indicating a synergistic effect of 2 µM SAG and 0.5 µM AKT inhibitor VIII on pituitary organoid induction. Hence, the sustained supplementation of 2 µM SAG and 0.5 µM AKT inhibitor VIII after day 40 was required (Figure 2C).

We juxtaposed the common condition and the initial condition to mimic the initial protocol reported in the literature.^[^ ^28^, ^29^ ^]^ Based on the results above, we formulated an improved protocol for pituitary organoids, amalgamating both common and improved conditions (Figure 2C). Immunofluorescence staining results demonstrated a substantially greater percentage of ACTH and PRL‐positive cells on day 100 in organoids cultured using the improved protocol compared to those obtained with the initial protocol (Figure 2D,E,G). Moreover, organoids cultured with the improved protocol exhibited markedly higher secretion levels of ACTH and PRL on day 100 compared to the initial protocol (Figure 2F,H). Additionally, qPCR analyses revealed significantly augmented relative expression levels of POMC and PRL mRNA on day 100 in organoids cultured with the improved protocol compared to the initial protocol (Figure 2I–J). Interestingly, after day 30, organoids cultured using the improved protocol exhibited smaller areas compared to those cultured with the initial protocol (Figure 2K; Figure S1D‐E, Supporting Information), a phenomenon potentially attributed to enhanced cellular differentiation and reduced proliferation within the improved protocol. These findings collectively highlight a significant improvement in the efficiency of pluripotent stem cell differentiation into pituitary organoids capable of secreting ACTH and PRL, facilitated by the optimized protocol.

### 2.3 Functional Evaluation of Pituitary Organoids Induced by the Improved Protocol

**Figure 3:**
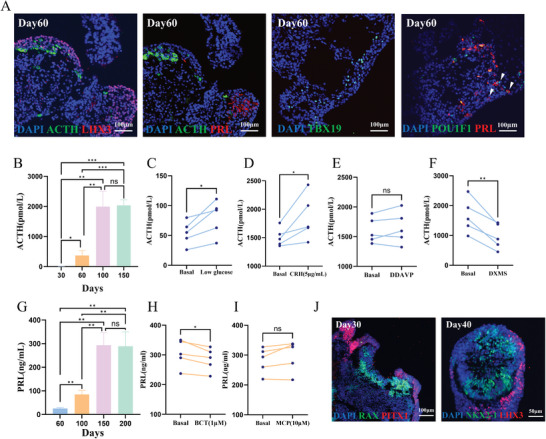
Functional evaluation of pituitary organoids induced by the improved protocol. A) Positive expression of LHX3, ACTH, PRL, TBX19, and POU1F1 in organoids on day 60, revealed by immunofluorescence staining (Scale bars: 100 µm). B) ACTH secretion levels of organoids on days 30, 60, 100, and 150 (N = 5, mean ± SD, Brown‐Forsythe and Welch's ANOVA test with post hoc Dunnett's T3 multiple comparisons test). C) Low glucose test (N = 5, Two‐tailed paired t‐test). D) CRH loading test (N = 5, Two‐tailed paired t‐test). E) DDAVP loading test (N = 5, Two‐tailed paired t‐test). F) DXMS loading test (DXMS: Dexamethasone, N = 5, Two‐tailed paired t‐test). G) PRL secretion levels of organoids on days 60, 100, 150, and 200 (N = 5, mean ± SD, Brown‐Forsythe and Welch's ANOVA test with post hoc Dunnett's T3 multiple comparisons test). H) BCT loading test (BCT: Bromocriptin, N = 5, Two‐tailed paired t‐test). I) MCP loading test (MCP: Metoclopramide, N = 5, Two‐tailed paired t‐test). J) RAX and PITX1 co‐expression within pituitary organoids on day 30 (Scale bars: 100 µm); NKX2‐1 and LHX3 co‐expression within pituitary organoids on day 40 (Scale bars: 50 µm). ns means no significance, ^*^ *p* < 0.05, ^**^ *p* < 0.01, and ^***^ *p* < 0.001.

Within the organoids cultured for 60 days using the improved protocol, we observed positive expression for key markers including LHX3, ACTH, PRL, TBX19 (T‐box transcription factor 19), and POU1F1 (POU class 1 homeobox 1) (**Figure** **3**A). Notably, our protocol facilitated the emergence of PRL‐positive cells as early as day 60, which is earlier than the reported onset at day 84 in prior literature.^[^ ^29^ ^]^ Additionally, while literature suggested that the ACTH secretion level stabilized around day 300 with an average value of ≈880 pmol L^−1^, our study demonstrated an initial increase followed by stabilization around day 100, indicating the earlier maturation of ACTH‐secreting cells within the organoids (Figure 3B).

Furthermore, literature indicated that the PRL secretion level stabilized around day 148 with a level of ≈50 ng mL^−1^.^[^ ^33^ ^]^ However, in our study, the PRL secretion level began to stabilize around day 150 (Figure 3G), with an average value of ≈293.3 ng mL^−1^, significantly surpassing the previously reported level. These findings underscore the substantial enhancement in hormone secretion levels achieved through the improved protocol, coupled with a shortened differentiation time.

In addition to assessing the baseline secretion levels of ACTH and PRL, we conducted functional tests to investigate whether ACTH or PRL secretion of the pituitary organoids could be regulated by alterations in culture medium. Functional tests for ACTH secretion conducted on organoids between days 100–150 yielded positive results for the low glucose test, the CRH stimulation test, and the dexamethasone suppression test, while the DDAVP test showed an increasing trend albeit being negative (Figure 3C–F). This successful replication of the CRH‐ACTH‐cortisol axis in vivo highlighted the functionality of the organoids. Similarly, PRL functional tests conducted on organoids from day 150 to day 200 demonstrated positive results for the bromocriptine suppression test, with the metoclopramide stimulation test showing an upward trend despite being negative (Figure 3H–I). These outcomes mirrored the regulatory effects of hypothalamic dopaminergic neurons on lactotrophs. Moreover, the positive outcome of the low glucose test suggested the presence of not only pituitary cells but also hypothalamic cells within the organoids. Detection of hypothalamic lineage markers RAX and NKX2‐1 on days 30 and 40 (Figure 3J), and detection of neuron markers MAP2 and NeuN on day 100 (Figure S1F–G, Supporting Information), further supported this notion, consistent with the previous reports.^[^ ^29^ ^]^ Collectively, these findings demonstrated that the pituitary organoids cultured using the improved protocol were capable of secreting ACTH and PRL, with their secretion regulated by varying conditions in the culture medium, thus affirming the success of the enhanced pituitary organoid induction protocol.

### 2.4 Cellular Diversity and Abundant Intercellular Interactions in Pituitary Organoids Revealed by scRNA‐Seq Analysis

**Figure 4:**
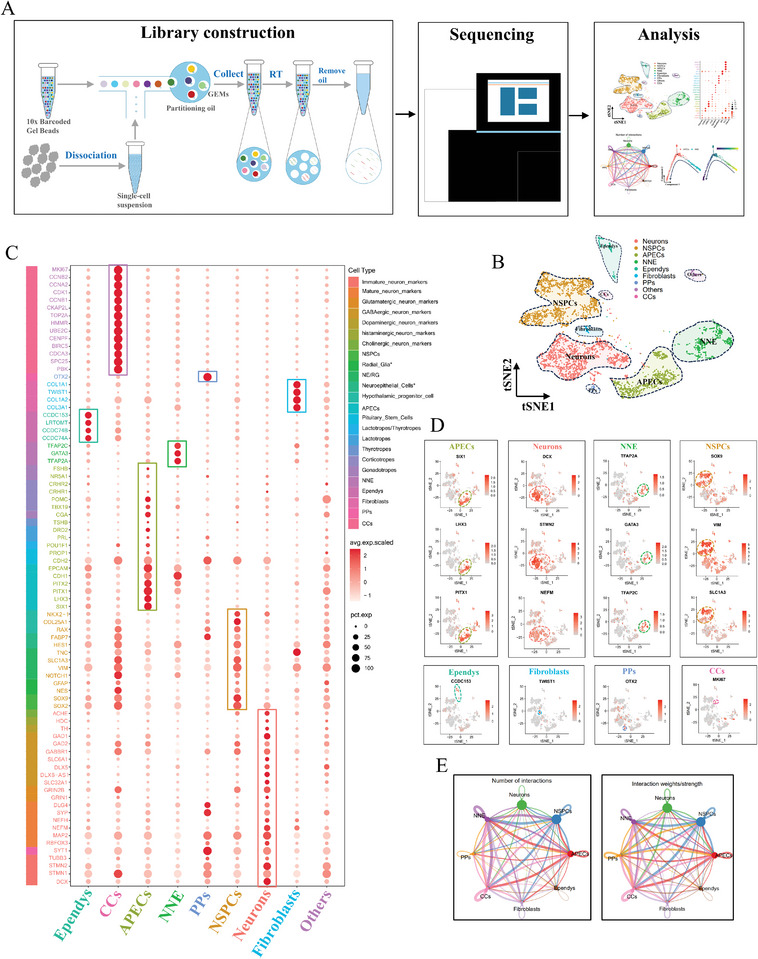
Cellular diversity and abundant intercellular interactions in pituitary organoids revealed by scRNA‐seq analysis. A) Schematic diagram of 10x Genomics scRNA‐seq. B) t‐SNE visualization of nine clusters within the sample of pituitary organoids. C) Display of specific genes in nine clusters using the dot plot, corresponding to Table S1 (Supporting Information). Dot size, pct.exp; dot color, avg.exp.scaled. D) Expression of known markers projected on the t‐SNE plot. APECs (anterior pituitary endocrine cells: SIX1, LHX3, PITX1); Neurons (DCX, STMN2, NEFM); NNE (non‐neural ectoderm: TFAP2A/C, GATA3); NSPCs (neural stem and progenitor cells: SOX9, VIM, SLC1A3); Ependys (ependymocytes: CCDC153); Fibroblasts (TWIST1), PPs (posterior pituicytes: OTX2); CCs (cycling cells: MKI67). Deeper red, higher expression. E) Network visualization of potential interactions between eight clusters. Nodes, clusters with specific colors; edge color, the same with the cluster where the ligand is located; edge width, numbers of the significant L‐R pairs between every two clusters (Left) or interaction strength between every two clusters (Right).

After a gradual dissociation process, single cell suspension was obtained from ten pituitary organoids and then subjected to high‐throughput labeling using the 10x Genomics platform followed by sequencing on the Illumina platform (**Figure** **4**A). After strict quality control as stated in the method, 4675 high‐quality cells were used for subsequent single‐cell analysis. An average of 4391 genes (nFeature_RNA) and 12 300 transcripts (nCount_RNA) were identified in each cell (Figure S2A, Supporting Information). Seurat was utilized for cell cluster identification, and t‐distributed Stochastic Neighbor Embedding (t‐SNE) was employed for visualization (Figure 4B). A total of nine cell clusters were identified (Figure S2B, Supporting Information; Figure 4C,D), including anterior pituitary endocrine cells (APECs, PITX1^+^LHX3^+^), neurons (DCX^+^STMN2^+^NEFM^+^), non‐neural ectoderm (NNE, TFAP2A/C^+^GATA3^+^), neural stem and progenitor cells (NSPCs, SOX9^+^VIM^+^SLC1A3^+^), ependymocytes (Ependys, CCDC153^+^), fibroblasts (TWIST1^+^), posterior pituicytes (PPs, OTX2^+^), cycling cells (CCs, MKI67^+^) and others (45 undefined cells).

Within the “Neurons” cluster, there were mature neurons characterized by MAP2 and NEFM, as well as immature neurons marked by DCX and STMN2 (Figure 4C; Table S1, Supporting Information). Additionally, this cluster encompassed diverse subtypes of neurons, such as glutamatergic, GABAergic, dopaminergic, histaminergic, and cholinergic neurons (Figure 4C; Table S1, Supporting Information). Cell cycle‐related markers, such as CDK1, CDCA3, CCNA1, CCNB2, were highly and widely expressed in cells of the “CC” cluster (Figure 4C; Table S1, Supporting Information).

Cell‐cell communication analysis was conducted on eight distinct clusters using CellChat,^[^ ^34^ ^]^ revealing abundant interactions between these clusters (Figure 4E; Figure S2C‐D and Table S2, Supporting Information). The interactions were mainly enriched in secreted signaling (TGFb, BMP, WNT, EGF, FGF pathways, etc.), ECM‐Receptor signaling (COLLAGEN, FN1, LAMININ pathways, etc.) and Cell‐Cell Contact signaling (NOTCH, CDH, NCAM pathways, etc.) (Figures S2C and S3A and Table S2, Supporting Information), which endowed pituitary organoids with significant advantages over cell lines in achieving complex physiological functions, maintaining structural integrity, and regulating cell fate determination.

### 2.5 Expression Patterns of Hypothalamic and Pituitary Lineage Markers in Pituitary Organoids Revealed by scRNA‐Seq Analysis

**Figure 5:**
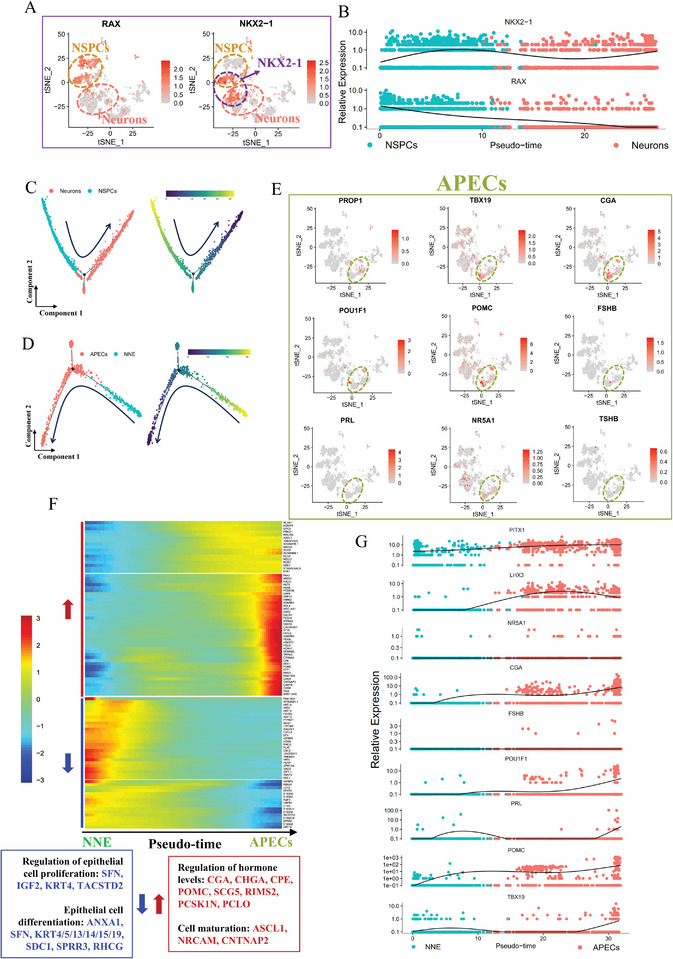
Expression patterns of hypothalamic and pituitary lineage markers in pituitary organoids revealed by scRNA‐seq analysis. A) Expression of hypothalamic lineage markers (RAX, NKX2‐1) projected on the t‐SNE plot. Deeper red, higher expression. B) Expression of RAX and NKX2‐1 along the pseudo‐time axis, with different colors representing different clusters. C) Development trajectory based on the integrated data from neurons and NSPCs. D) Development trajectory based on the integrated data from APECs and NNE. E) Expression of pituitary lineage markers (PROP1, POU1F1, PRL, TBX19, POMC, NR5A1, CGA, FSHB, TSHB) projected on the t‐SNE plot. Deeper red, higher expression. F) Heatmap displays the top 100 differentially expressed genes along the pseudo‐time axis. Each row represents a gene, and the horizontal axis represents the pseudo‐time axis. Blue represents genes with decreased expression along the pseudo‐time axis, while red represents genes with increased expression along the pseudo‐time axis. G) Expression of pituitary lineage markers (PROP1, POU1F1, PRL, TBX19, POMC, NR5A1, CGA, FSHB, TSHB) along the pseudo‐time axis, with different colors representing different clusters.

Previous studies indicated that NKX2‐1 was expressed in both early progenitor cells^[^ ^35^ ^]^ and late‐stage neuroendocrine cells^[^ ^36^, ^37^ ^]^ during the development of hypothalamic neurons, whereas RAX was mainly expressed in early progenitor cells.^[^ ^32^ ^]^ Our scRNA‐seq analysis showed that RAX was primarily expressed in the NSPCs, while NKX2‐1 was expressed in both NSPCs and neurons (**Figure** **5**A). Additionally, upon integrating the NSPCs and neurons for developmental trajectory analysis (Figure 5C), we observed a continuous decrease in RAX expression along the pseudo‐time axis, whereas NKX2‐1 exhibited an initial increase followed by a slight decline before ultimately rising (Figure 5B), consistent with the expression trends observed in the organoids during development (Figure S5E, Supporting Information). Furthermore, along the pseudo‐time axis, the expression levels of NSPC markers VIM and SLC1A3 decreased, while the neuron marker DCX increased (Figures S3B and S5E, Supporting Information). These findings indicated that the expression patterns of hypothalamic lineage markers in pituitary organoids were consistent with previous research, which provided a reliable foundation for exploring the interaction between the hypothalamus and pituitary using the pituitary organoid model.

Corticotrope markers POMC and TBX19 were detected in 32.875% and 14.75% of APECs, respectively (Figures 4C and 5E; Table S1, Supporting Information), indicating that corticotropes comprised the predominant cell type of APECs. The scRNA‐seq analysis revealed APECs and NNE as the predominant clusters within the non‐neural lineage. Given that APECs originated from NNE, we integrate the two clusters for developmental trajectory analysis (Figure 5D). Notably, there was a discernible increase in the expression of POMC and TBX19 along the pseudo‐time axis (Figure 5G). Luteinizing hormone (LH), follicle stimulating hormone (FSH), and thyroid stimulating hormone (TSH) share the same α‐subunit (CGA), but their β‐subunits are different, namely LHB, FSHB, and TSHB.^[^ ^38^ ^]^ CGA was detected in 31% of APECs, while LHB, FSHB, and TSHB were only found in 0.5%, 1.25%, and 0.25% of APECs, respectively (Figures 4C and 5E; Table S1, Supporting Information). This result suggested that the majority of CGA^+^ cells failed to express the genes for the β‐subunits, underscoring a significant concern that should be considered in future protocol optimization endeavors. POU1F1 was found to be expressed in 10.875% of APECs, while PRL, TSHB, and GH1 were expressed in only 1.625%, 0.25%, and 0.125% of APECs, respectively (Figures 4C and 5E; Table S1, Supporting Information). This result indicated the existence of a small cell subset, which was defined as Pro.PIT1 by Zhang et al.^[^ ^39^ ^]^ Gonadotrope markers NR5A1, FSHB, and LHB were detected in 5.875%, 1.25%, and 0.5% of APECs (Figures 4C and 5E; Table S1, Supporting Information), respectively, indicating the presence of a small cell subset identified by Zhang et al. as Pre. Gonado.^[^ ^39^ ^]^ Notably, the expression levels of CGA, CHGB,^[^ ^40^ ^]^ POU1F1, and PRL increased along the pseudo‐time axis (Figure 5F,G).

Furthermore, we used Metascape to conduct enrichment analysis on genes that either increased or decreased along the pseudo‐time axes. Along the NNE‐APECs pseudo‐time axis, genes that decreased were enriched in biological processes related to epithelial cell proliferation and differentiation (Figure 5F; Table S3, Supporting Information), aligning with the anterior pituitary's origin from oral ectodermal epithelium.^[^ ^41^ ^]^ In contrast, genes that increased were enriched in processes associated with hormone regulation and cell maturation (Figure 5F; Table S3, Supporting Information). For the NSPCs‐Neurons pseudo‐time axis, genes that decreased were primarily enriched in biological processes related to neural precursor cell proliferation and embryonic organ development (Figure S3B and Table S3, Supporting Information). Conversely, genes that increased were associated with cellular components such as presynapses, postsynapses, neuronal cell bodies, axons, and dendrites, as well as biological processes like trans‐synaptic signaling (Figure S3B and Table S3, Supporting Information). These findings provided valuable insights into the dynamic transcriptional changes underlying the differentiation and maturation of pituitary and neuronal cells, highlighting distinct developmental trajectories and the biological processes that drive lineage‐specific specialization.

Collectively, scRNA‐seq analysis elucidated the cellular composition of the pituitary organoids, laying a strong groundwork for future research on protocol optimization and application studies.

### 2.6 Spatial Transcription of the Pituitary Organoid Revealed by Stereo‐Seq Analysis

**Figure 6:**
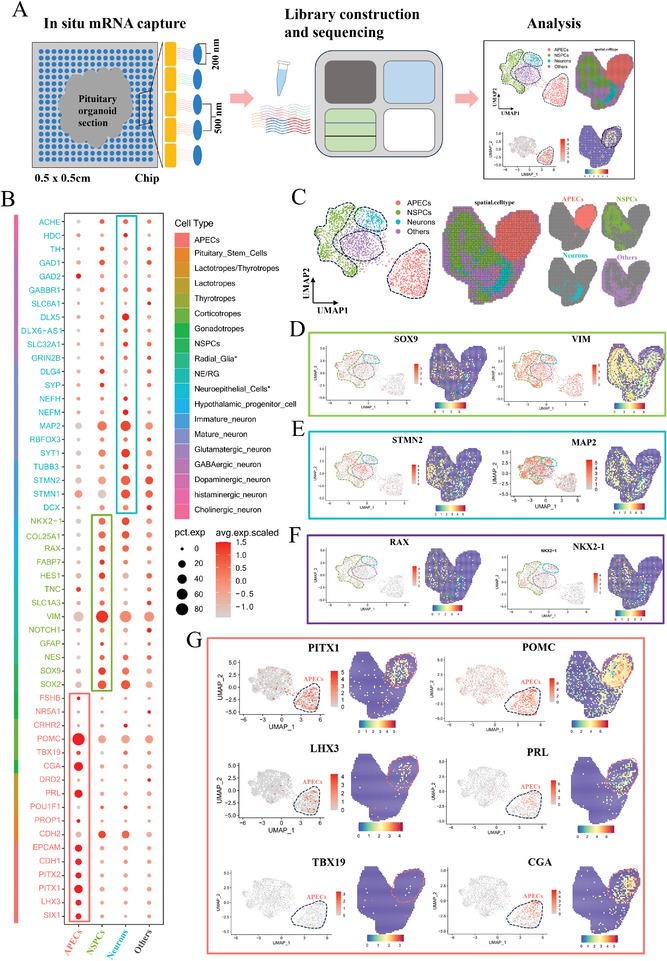
Spatial transcription of the pituitary organoid revealed by Stereo‐seq analysis. A) Schematic diagram of Stereo‐seq analysis. B) Display of specific genes in four clusters using the dot plot. Dot size, pct.exp; dot color, avg.exp.scaled. C) UMAP visualization and spatial visualization of four clusters. D) Expression of SOX9 and VIM projected on the UMAP plot and their spatial distribution. E) Expression of STMN2 and MAP2 projected on the UMAP plot and their spatial distribution. F) Expression of RAX and NKX2‐1 projected on the UMAP plot and their spatial distribution. G) Expression of APECs markers (PITX1, LHX3, TBX19, POMC, PRL, CGA) projected on the UMAP plot and their spatial distribution.

Fresh pituitary organoids were embedded in O.C.T. and immediately frozen, followed by frozen section preparation, permeabilization, library construction, sequencing, and subsequent data analysis (**Figure** **6**A). The correlation coefficient of the Stereo‐seq data reached 0.99, indicating high‐quality library construction and that the cells in the library were in good condition (Figure S4A, Supporting Information). Four cell clusters were identified (Figure 6B,C), namely APECs (POMC^+^CGA^+^PRL^+^, Figure 6G), neurons (STMN2^+^MAP2^+^, Figure 6E), NSPCs (VIM^+^ SOX9^+^, Figure 6D), and others classified as low‐quality data (Figure S4B‐C, Supporting Information). In contrast to scRNA‐seq results, Stereo‐seq results demonstrated fewer clusters, more expression of POMC (81.772% versus 32.875%), CGA (33.731% versus 31%), and PRL (26.065% versus 1.625%) in APECs (Figure 6G; Table S4, Supporting Information), and fewer expression of transcription factors such as TBX19 (1.363% versus 14.75%), POU1F1 (0.17% versus 10.875%), and NR5A1 (0 versus 5.875%) (Figure S4D and Table S4, Supporting Information). Hypothalamic marker NKX2‐1 was detected in 22.5% of neurons and 15.1% of NSPCs (Figure 6F; Table S4, Supporting Information).

Most importantly, Stereo‐seq provided spatial information of specific cell types that was not attainable by scRNA‐seq. The pituitary organoid exhibited an irregular shape, characterized by the aggregation of APECs resembling the structure of the pituitary gland. The hypothalamic and neural lineage cells were observed to cluster together, reminiscent of the feature seen in the hypothalamic region. Additionally, non‐neural lineage APECs were spatially adjacent to neural lineage neurons and NSPCs (Figure 6C). Considering the abundant intercellular interactions between APECs, neurons, and NSPCs (Figure 4E; Figure S2C‐D, Supporting Information), the pituitary organoids might serve as an ideal experimental model for investigating the mechanisms underlying hypothalamus‐pituitary interactions.

### 2.7 SOX3/SHH Signaling Pathway was Crucial for Inducing the LHX3 Expression of Pituitary Organoids

**Figure 7:**
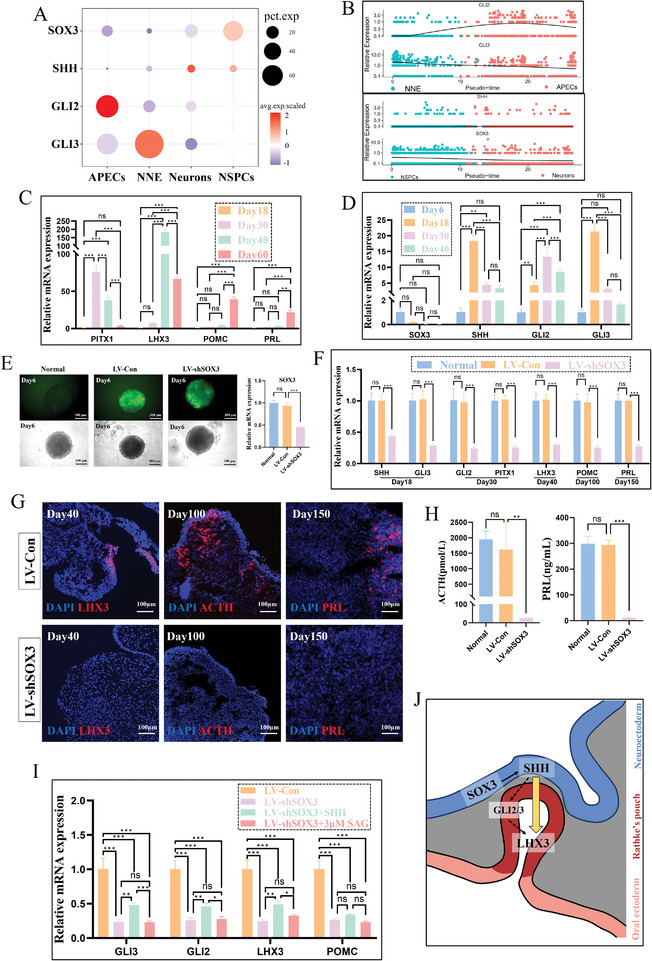
SOX3/SHH signaling pathway was crucial for inducing the LHX3 expression of pituitary organoids. A) Display of specific genes (SOX3, SHH, GLI2, GLI3) in different clusters (APECs NNE, Neurons, NSPCs) using the dot plot. Dot size, pct.exp; dot color, avg.exp.scaled. B) Expression of specific genes (SOX3, SHH, GLI2, GLI3) along the pseudo‐time axis, with different colors representing different clusters. C–D) Expression of PITX1, LHX3, POMC, PRL, SOX3, SHH, GLI2, GLI3 mRNA in organoids cultured for different days (N = 3, mean ± SD, two‐way ANOVA test with post hoc Bonferroni's method). E) The expression of SOX3 in the organoids was successfully suppressed to below 50% through two rounds of lentivirus transfection (N = 3, mean ± SD, one‐way ANOVA test with post hoc Bonferroni's method). F) Impact of SOX3 gene interference on the mRNA expression levels of SHH, GLI2, GLI3, PITX1, LHX3, POMC, and PRL in organoids (N = 3, mean ± SD, two‐way ANOVA test with post hoc Bonferroni's method). G) Impact of SOX3 gene interference on the expression of LHX3 (day40), POMC (day100), and PRL (day150) within organoids, revealed by immunofluorescence staining (Scale bars: 100 µm). H) Impact of SOX3 gene interference on the ACTH and PRL secretion levels of organoids (N = 5, mean ± SD, one‐way ANOVA test with post hoc Bonferroni's method). I) Relative expression levels of GLI3, GLI2, LHX3, and POMC mRNA in four groups (N = 5, mean ± SD, two‐way ANOVA test with post hoc Bonferroni's method). J) Schematic image of the role of SOX3/SHH signaling in pituitary development. SHH, secreted by SOX3‐positive neural lineage cells, plays a critical role in facilitating the expression of rathke's pouch marker LHX3. ns means no significance, ^*^ *p* < 0.05, ^**^ *p* < 0.01, and ^***^ *p* < 0.001.

Our scRNA‐seq analysis revealed that SOX3 exhibited predominant expression in NSPCs, while SHH was expressed in NSPCs and neurons at a lower proportion (**Figure** **7**A). Along the pseudo‐time axis, the expression of SOX3 demonstrated a consistent decline, whereas SHH expression remained relatively stable (Figure 7B), possibly attributable to the limited presence of SHH‐positive cells in NSPCs and neurons. GLI2 and GLI3, downstream signaling molecules of SHH, displayed predominant expression in non‐neural lineages. More precisely, GLI2 demonstrated predominant expression in APECs with an increasing trend along the pseudo‐time axis, while GLI3 was primarily expressed in NNE with a progressive decrease along the pseudo‐time axis (Figure 7A,B). We further explored the mRNA expression of SHH, GLI2, GLI3, PITX1, and LHX3 in the pituitary organoids by quantitative PCR experiments, and detected their peaks at day 18, 30, 18, 30, and 40, respectively (Figure 7C,D).

The expression of SOX3 in the organoids was successfully suppressed to below 50% through two rounds of lentivirus transfection (Figure 7E). In comparison to LV‐Con group, the organoids of LV‐shSOX3 group exhibited notable decreases in the expression levels of SHH, GLI2, GLI3, PITX1, LHX3, POMC, and PRL (Figure 7F,G). Additionally, the POMC and PRL secretion levels of the pituitary organoids in the LV‐shSOX3 group were significantly diminished (Figure 7H). These findings suggested that gene interference targeting SOX3 effectively impeded the development and differentiation of the pituitary organoids.

To investigate whether the sonic hedgehog signaling pathway was involved in the developmental regulation of pituitary organoids by SOX3, recombinant protein SHH was continuously supplemented at a final concentration of 200 ng mL^−1^ after day 6. The results showed that the supplementation of recombinant protein SHH partially rescued the decrease in GLI2, GLI3, and LHX3 expression caused by SOX3 gene interference (Figure 7I). However, no rescue effect was observed in the group supplemented with an additional 3 µm SAG, implying the pivotal involvement of the SMO‐independent signaling cascade in pituitary development (Figure 7I). In conclusion, SHH, secreted by SOX3‐positive neural lineage cells, might play a critical role in facilitating the differentiation of non‐neural lineage cells into the pituitary (Figure 7J).

## 3 Discussion

### 3.1 Three Advancements of Our Research

Our work has yielded three advancements in the field of pituitary organoid research. First, through systematic adjustments in culture conditions, we successfully enhanced the efficiency of iPSCs’ differentiation into human pituitary organoids, as evidenced by increased expression of key markers and hormone secretion. Second, scRNA‐seq and Stereo‐seq analyses revealed the diverse cellular composition and abundant intercellular interactions within the organoids, providing valuable insights into their structural and functional complexity. Thirdly, our findings also indicated that SHH secreted by SOX3‐positive neural lineage cells played a crucial role in pituitary organoid development, as evidenced by the diminished levels of key markers and hormone secretion upon SOX3 gene interference, which could be partially rescued by recombinant SHH protein supplementation. Collectively, these advancements contributed to a deeper comprehension of the structure and function of pituitary organoids, and lay the groundwork for investigating the pathogenesis and therapeutic strategies for human pituitary diseases.

### 3.2 Advancements and Challenges in Pituitary Organoid Culture Protocols

The existing culture protocols for 3D pituitary organoids or hypothalamus‐pituitary organoids^[^ ^29^, ^42^, ^43^ ^]^ were developed through incremental adjustments to the culture protocols for 3D hypothalamus organoids.^[^ ^32^ ^]^ The 3D pituitary organoids or hypothalamus‐pituitary organoids typically consist of non‐neural lineage cells or pituitary cells in the outer layer and neuronal cells or hypothalamic neurons in the inner layer. Based on a comprehensive review of the literature and insights from developmental biology,^[^ ^29^, ^30^, ^32^, ^43^, ^44^, ^45^ ^]^ we speculate that signaling molecules such as BMP4 and FGF2 might facilitate the differentiation of outer layer cells within organoids toward pituitary lineage cells, while the inner layer cells of organoids tend to differentiate into neural lineage cells due to lower concentrations of signaling molecules encountered by the inner layer. The difference of signaling molecule concentrations encountered by the inner and outer layer cells within organoids or spheroids may be the logical foundation for the construction of hypothalamus‐pituitary organoids. Our systematic adjustments to the culture protocol for pituitary organoids have resulted in a successful enhancement of iPSCs’ differentiation into pituitary organoids, as evidenced by the notable increase in key biomarkers and hormone secretion levels. Nevertheless, the persistence of non‐pituitary lineage cells remains a prevalent challenge in current 3D pituitary organoid models.^[^ ^46^ ^]^ Actually, the presence of non‐pituitary lineage cells may be a double‐edged sword, considering their removal through purification is required before transplantation to patients with hypopituitarism.^[^ ^47^, ^48^ ^]^ From a positive perspective, the inclusion of non‐pituitary lineage cells and the intricate intercellular interactions between non‐pituitary and pituitary lineage cells within pituitary organoids offer a valuable opportunity to explore the complex mechanisms of the hypothalamic‐pituitary axis,^[^ ^28^ ^]^ which renders pituitary organoids a promising laboratory model for the investigation of human pituitary diseases. Our study demonstrated the significance of SHH secretion by SOX3‐positive neural lineage cells in pituitary development based on scRNA‐seq analysis and pituitary organoid models, which validated the usefulness of pituitary organoids as a valuable experimental model for investigating human pituitary diseases and provided a research template and accessible transcriptomic datasets for future exploration in this field.

### 3.3 Integration and Comparative Analysis of scRNA‐Seq and Stereo‐Seq in Pituitary Organoids

The integration of scRNA‐seq and Stereo‐seq is of great significance for elucidating the cellular heterogeneity, intercellular communication, and spatial distribution within pituitary organoids. Our scRNA‐seq analysis identified distinct cell clusters within pituitary organoids, including rare cell types such as ependymocytes, fibroblasts, posterior pituicytes, and cycling cells, leading to a more comprehensive insight into the cellular composition and functional properties of pituitary organoids. However, scRNA‐seq fails to provide spatial information of specific cell types within pituitary organoids, which can be provided by Stereo‐seq.^[^ ^15^, ^16^ ^]^ As a high‐throughput, high‐resolution in situ panoramic technique developed based on DNA Nano Balls DNBs, Stereo‐seq enables the elucidation of spatial distribution and relative positional relationships among different cell types within pituitary organoids, thus promoting the understanding of intercellular interactions and signal transduction pathways within pituitary organoids. In conclusion, the integration of scRNA‐seq and Stereo‐seq overcomes the constraints of each technique, enabling a more comprehensive analysis of pituitary organoids. This in‐depth understanding of pituitary organoids lays a solid groundwork for future research endeavors focused on the applications of pituitary organoids, as well as pathogenesis and therapeutic interventions for human pituitary diseases.

Stereo‐seq and scRNA‐seq exhibited variances in the analysis of pituitary organoids. In contrast to scRNA‐seq, Stereo‐seq showed limited sensitivity in the detection of transcription factors such as TBX19, POU1F1, and NR5A1 (Figure S4D, Supporting Information), possibly due to their lower abundance or expression levels. Additionally, discrepancies existed between the levels of transcripts corresponding to cell surface proteins and their actual distribution levels, suggesting caution in interpreting the data. Our scRNA‐seq results indicated the presence of CRH receptors on the surface of POMC‐positive cells, particularly CRHR1 rather than CRHR2 (Figure S5C‐D, Supporting Information), in line with prior research.^[^ ^29^ ^]^ However, scRNA‐seq data showed that only a minority of POMC‐positive cells expressed CRHR1 (Figure S5C,F, Supporting Information), and the application of Stereo‐seq did not identify CRHR1 expression (Figure S5D, Supporting Information), contradicting the established biological knowledge. Consistent with the positive results of the bromocriptine inhibition test, scRNA‐seq results demonstrated the existence of dopaminergic neurons and DRD2^+^ lactotrophs within pituitary organoids (Figure S5B, Supporting Information). Nevertheless, Stereo‐seq failed to identify DRD2 expression in lactotrophs (Figure S5B, Supporting Information), contrary to the scRNA‐seq results and established biological knowledge.^[^ ^49^ ^]^ Interestingly, our sequencing results revealed that EPCAM was predominantly expressed in APECs, while CDH1 was highly expressed in both APECs and NNE clusters (Figure S5A, Supporting Information), suggesting that EPCAM might be a more viable marker for cell purification of pituitary organoids, in accordance with the prior studies.^[^ ^47^, ^48^ ^]^ In conclusion, the interpretation of scRNA‐seq and Stereo‐seq data should be complemented by a thorough review of existing literature and biological understanding.

### 3.4 Comparison of 3D Pituitary Organoids and 2D Pituitary Cells derived from iPSCs

As previously mentioned, 3D pituitary organoids, characterized by their diverse cell types and abundant intercellular communication, may offer a more suitable platform for investigating pathogenesis in comparison to 2D pituitary cells. Conversely, pituitary cells derived from iPSCs in a 2D environment seem to be more suitable for regenerative medicine therapy in patients with hypopituitarism.^[^ ^30^, ^45^ ^]^ It is noteworthy that our study revealed that SAG could not fully replace SHH secreted by non‐pituitary lineage cells or recombinant protein SHH added to the culture medium, implying the critical involvement of the SMO‐independent signaling cascade in pituitary development.^[^ ^50^ ^]^ This finding suggests that supplementation with recombinant protein SHH may be crucial for iPSCs’ differentiation into pituitary cells in a 2D environment.

### 3.5 Application of Pituitary Organoids

In clinical, mutations or defects in SOX3 gene have been linked to congenital pituitary hypoplasia and CPHD.^[^ ^21^, ^22^, ^23^ ^]^ However, the precise role of SOX3 in human pituitary development remains incompletely understood due to the inherent limitations of murine models. Our study addresses this gap by employing human iPSCs‐derived pituitary organoids to examine the effects of SOX3 gene interference on pituitary development. The findings revealed that SHH, secreted by SOX3‐positive neural lineage cells, might play a critical role in facilitating the differentiation of non‐neural lineage cells into pituitary cells. The observed reduction in ACTH secretion of SOX3‐interfered organoids parallels the ACTH deficiencies observed in patients with SOX3 gene mutations,^[^ ^21^, ^22^, ^23^ ^]^ underscoring the clinical relevance of our findings. This study validated the utility of pituitary organoids as a robust experimental model for investigating human pituitary disorders. Furthermore, these insights may offer potential avenues for therapeutic development, with the SMO‐independent SHH signaling pathway emerging as a promising therapeutic target for patients with SOX3 gene mutations.

In addition to advancing the understanding of human pituitary development and the pathogenesis of genetic pituitary disorders, pituitary organoids present substantial potential for investigating acquired pituitary diseases. Keitaro et al. utilized human iPSC‐derived pituitary organoids to explore the pathogenesis of anti‐PIT‐1 hypophysitis.^[^ ^51^ ^]^ Furthermore, a prospective review emphasized that integrating gene editing technologies with pituitary organoid models could provide novel insights into the pathogenesis of pituitary adenoma.^[^ ^52^ ^]^ Several studies have demonstrated that subcapsular or subcutaneous transplantation of pituitary organoids into mice could enhance serum hormone levels and survival rates in pituitary‐deficient models,^[^ ^42^, ^43^, ^45^, ^47^ ^]^ highlighting the regenerative potential of pituitary organoids for treating patients with pituitary dysfunction. With the continuous development of pituitary organoid technology, this model will provide innovative strategies for drug screening and therapeutic interventions of pituitary‐related diseases.

### 3.6 Limitations of Our Study

Nonetheless, there are several limitations in our study. First, corticotrophs and lactotrophs dominate the APECs cluster of the pituitary organoids, while somatotrophs, thyrotrophs, and gonadotrophs are notably absent. The overrepresentation of corticotrophs and lactotrophs may reflect potential biases in the differentiation pathways during organoid development, possibly due to limitations in the differentiation protocols or specific microenvironmental cues provided to the stem cells. Future research should explore alternative methods, such as co‐culture systems or the introduction of specific growth factors, to promote a more diverse population of pituitary cells. Additionally, due to budget constraints, we were unable to perform sequencing of the pituitary organoids at different time points. This limitation may affect our in‐depth understanding of the dynamic differentiation of cell types and their biological significance. Addressing these issues will not only enhance the validity of our findings but also advance the development of more representative pituitary organoid models for studying endocrine regulation and pituitary‐related diseases.

## 4 Conclusion

To our knowledge, this is the first study to decode the transcriptional landscape of pituitary organoids by scRNA‐seq and Stereo‐seq, as well as the first study to investigate the role of SOX3/SHH signaling on pituitary development using the human organoid model. This study provides not only an accessible and minable transcriptomic dataset for future explorations, but also a research paradigm for subsequent endeavors in this field.

## 5 Experimental Section

### Human iPSCs

Human iPSCs (DYR0100) were established from the foreskin cells of healthy male newborns and kindly provided by Stem Cell Bank, Chinese Academy of Sciences. All reagents used for iPSCs’ culture were detailed in Table S5 (Supporting Information). Human iPSCs were cultured on Matrigel‐coated plates in mTeSR1 complete culture medium. Passaging was conducted at intervals of 4–7 days, with split ratios ranging from 1:10 to 1:20, utilizing a dissociation solution containing 0.02% EDTA. On the first day after resuscitation, human iPSCs displayed a spindle‐shaped morphology due to the addition of Y27632. Starting from the second day, Y27632 was no longer added to the culture medium, leading to a gradual reversion of human iPSCs to a clone‐like morphology (Figure S5G, Supporting Information). On the 5th to 7th day after resuscitation, the confluence of clones formed by human iPSCs reached ≈70%, characterized by dense central regions and well‐defined boundaries. Immunofluorescence staining revealed that a majority of cells within the clone exhibited elevated levels of pluripotency markers NANOG and OCT4 (Figure S5H, Supporting Information), suggesting the robust maintenance of pluripotency in human iPSCs, which served as a fundamental prerequisite for subsequent experiments.

### The Improved Culture Protocol for 3D Human Pituitary Organoids

All reagents used for organoids’ culture were detailed in Table S5 (Supporting Information). The composition of Pituitary Organoid Culture Medium (POCM) included growth factor‐free Chemically Defined Medium (gfCDM),^[^ ^32^ ^]^ a specified concentration of KSR, and precise levels of signaling pathway activators or inhibitors. Stage 1 (Day 0 to Day 3): POCM1 comprised gfCDM, 7.5% (vol/vol) KSR, 20 µM Y27632, 100 nM LDN‐193189, and 100 nM SB431542. Stage 2 (Day 3 to Day 6): POCM2 included gfCDM, 7.5% (vol/vol) KSR, 100 nM SB431542. Stage 3 (Day 6 to Day 18): POCM3 consisted of gfCDM, 7.5% (vol/vol) KSR, 7.5 nM BMP4, 2 µM SAG, 100 nM SB431542. Stage 4 (Day 18 to Day 30): POCM4 was formed from gfCDM, 10% (vol/vol) KSR, 2 µM SAG, 50 ng mL^−1^ FGF2. Stage 5 (Day 30 to Day 60): POCM5 was gfCDM, 15% (vol/vol) KSR, 2 µM SAG.Stage 6 (Day 60 onwards): POCM6 included gfCDM, 20% (vol/vol) KSR, 2 µM SAG.

Human iPSCs were dissociated into single cells using Accutase once they reached 70–85% confluence. Cells were seeded into 96‐well V‐bottom ultra‐low attachment microplates at a density of 5000 cells per 100 µL POCM1 per well. By Day 3, cells spontaneously aggregated into spheroids, at which point POCM1 was entirely replaced with 100 µL POCM2 per well. From Day 6 to Day 18, POCM2 was completely replaced with 100 µL POCM3 per well, with medium changes every three days. Starting from Day 18, the organoids were relocated to an incubator set at 40% O_2_, 5% CO_2_. On Day 18, POCM3 was retained with 100 µL POCM4 being added to each well. For stage 4, 100 µL old medium was removed from each well every three days and replaced with 100 µL fresh POCM4. For stage 5, POCM4 was substituted with 200 µL POCM5 per well, with complete medium replacement every three days. For stage 6, POCM5 was exchanged for 200 µL POCM6 per well, with complete medium replacement every two days. Notably, the organoids were cultured in 96‐well V‐bottom ultra‐low attachment microplates throughout the entire process, without using Matrigel.

### Quantitative PCR

Sufficient samples of organoids were collected and subjected to RNA extraction utilizing the TRIzol method. The extracted RNA was subsequently reverse‐transcribed to cDNA according to the manufacturer's instructions for HiScript III All‐in‐one RT SuperMix. qPCR was conducted using Taq Pro Universal SYBR qPCR Master Mix and FTC3000 system. Data normalization was performed using the corresponding levels of ACTB mRNA as a reference, and the relative expression levels of the target genes were determined using the 2^‑ΔΔCT^ method. The primer sequences were detailed in Table S5 (Supporting Information).

### Immunostaining

The organoids were gently washed three times with DPBS solution and fixed with 4% paraformaldehyde for approximately 15 to 30 minutes, followed by overnight dehydration in 25% sucrose solution. Subsequently, the dehydrated organoids were embedded in OCT compound and quickly frozen at −80 °C. The organoids were sliced into 10 µm sections, and the resulting sections were meticulously affixed to glass slides. Following permeabilization and blocking using immunostaining blocking buffer, the sections were incubated with the primary antibody overnight at 4 °C, followed by five washes with DPBS solution for five minutes each. Subsequently, the sections underwent a 30‐minute incubation with secondary antibodies at room temperature, followed by five washes for five minutes each. The organoid sections were treated with DAPI mounting medium, covered with coverslips, and subsequently stored in darkness at 4 °C. Fluorescence images were acquired utilizing Zeiss LSM700 confocal microscopy. All antibodies are detailed in Table S5 (Supporting Information).

### ACTH Measurement in Steady State

On the 29th, 59th, 99th, and 149th days of organoid cultivation, the culture medium was completely replaced. Following 24 h of continuous incubation at 37 °C, the supernatant was collected. The supernatant from every 10 organoids was mixed into one sample. At least five samples were measured at each time point, with fresh medium serving as the blank control. The ACTH concentration was determined using a clinical‐grade ACTH assay kit. The ACTH secretion level of the organoids was determined by calculating the discrepancy in ACTH concentration between the organoid supernatant and the fresh medium.

### Low Glucose Test

The low glucose culture medium specifically for low glucose test, containing 100 mg L^−1^ glucose, was prepared by mixing low‐glucose DMEM containing 1000 mg L^−1^ glucose with no‐glucose DMEM at a ratio of 1:9. Organoids from day 100 to day 120 were subjected to low glucose test. The culture medium of 10 organoids was replaced with the low‐glucose DMEM, and after 40 min, the supernatant was collected. Subsequently, the medium was replaced with the prepared low‐glucose medium containing 100 mg L^−1^ glucose, and after another 40 min, the supernatant was collected again. The ACTH concentration in the supernatant was quantified, with no‐glucose DMEM serving as the blank control. A pair of supernatant samples before and after low glucose treatment was collected from every 10 organoids, with at least five pairs of samples collected.

### CRH/Dexamethasone/DDAVP Loading Test

Organoids from day 100 to day 150 were subjected to these three loading tests. The culture medium for 10 organoids was completely substituted with POCM6, followed by a 24‐hour incubation at 37 °C, after which the supernatant was collected. Subsequently, POCM6 supplemented with 5 µg mL^−1^ recombinant CRH protein, or 500ng mL^−1^ dexamethasone, or 1 µM DDAVP was then added, and after another 24‐hour incubation, the supernatant was collected once more. The ACTH concentration in the supernatant was quantified, with POCM6 serving as the blank control. For every 10 organoids, a pair of samples before and after the treatment was collected. At least five pairs of samples were obtained.

### PRL Measurement in Steady State

The culture medium was completely replaced on the 59th, 99th, 149th, and 199th days of organoid cultivation, followed by a 24‐hour incubation at 37 °C to collect the supernatant. Subsequently, the supernatant from every 10 organoids was mixed into one sample. A minimum of five samples were assessed at each time point using a clinical‐grade PRL assay kit to quantify the PRL secretion level of the organoids.

### Metoclopramide/Bromocriptine Loading Test

Organoids from day150 to day200 were subjected to the two loading tests. The culture medium for 10 organoids was completely substituted with POCM6, followed by a 24‐hour incubation at 37 °C, after which the supernatant was collected. Subsequently, POCM6 supplemented with either 1 µM Bromocriptine or 10 µM Metoclopramide was then added, and after another 24 h of continuous incubation, the supernatant was collected again. For every 10 organoids, a pair of samples before and after the treatment were collected. At least five pairs of samples were obtained.

### Preparation of Single‐Cell Suspensions from Organoids

Ten pituitary organoids cultured for 150 days, were harvested and finely minced into fragments. The fragments were then digested in DMEM/F12 medium supplemented with 0.1% BSA, 20 µM Y27632, 2 mg mL^−1^ collagenase I, and 1 mg mL^−1^ neutral protease at 37 °C for 30 min on a shaker. Following centrifugation, the supernatant was decanted and the cell precipitation was resuspended in 1xTryPLE dissociation solution supplemented with 0.2 mg mL^−1^ DNaseI and 20 µM Y27632. The suspension was then incubated at 37 °C for 10 min on a shaker, followed by gentle pipetting and passage through a 40 µm cell filter. Subsequently, the supernatant was removed by centrifugation, and the cell precipitation was resuspended in DPBS containing 0.04% BSA for subsequent single‐cell sequencing.

### scRNA‐Seq Library Preparation and Sequencing

As stated in the previous literature,^[^ ^15^, ^53^ ^]^ the 10x Genomics Chromium platform was utilized to encapsulate single cell and individual gel bead within a water‐in‐oil droplet, resulting in the formation of Gel Bead‐in‐Emulsions (GEMs). After cell lysis, mRNA was released and reversely transcripted to cDNA within the GEM reaction system. Subsequently, the cDNA was amplified, and libraries were constructed. High‐quality libraries were then sequenced using the Illumina platform with the PE150 strategy to obtain single‐cell transcriptome raw data.

### scRNA‐Seq Data Analysis and Visualization

The raw data were processed by Cell Ranger software (10x Genomics), followed by annotation through alignment to the GRCh38 genome reference.^[^ ^54^ ^]^ 8163 cells were captured. Cells with >20% mitochondrial counts, or unique feature counts (nFeature_RNA) over 10 000 or less than 500 were filtered by Seurat. After cell filtration, the remaining 8135 cells were divided into 21 clusters by Seurat software,^[^ ^55^ ^]^ and cluster 1 and 2 were removed using the subset function because there were mainly background impurities and no valuable cells in the two clusters. The downstream analyses including clustering, marker identification, pseudotime analysis and cell‐cell interaction analysis were based on data of the remaining 4675 high‐quality cells. Cell clustering analysis was conducted utilizing the Seurat software and visualized using t‐Distributed Stochastic Neighbor Embedding (t‐SNE) technology, resulting in the identification of nine major cell populations. Pseudotime analysis was performed using Monocle software on neural lineage cells and non‐neural lineage cells separately.^[^ ^56^ ^]^ The CellChat was used to identify receptor‐ligand pairs among clusters,^[^ ^34^ ^]^ indicating potential interactions or communication between them.

### Stereo‐Seq Library Preparation and Sequencing

The pituitary organoid cultured until day 150 was embedded in O.C.T. and rapidly frozen at −80 °C to maintain tissue structure and RNA integrity. The library construction for Stereo‐seq was performed by Annoroad Gene Technology, in accordance with the previously published protocol.^[^ ^16^ ^]^ The 10 µm frozen sections were affixed to Stereo‐seq TO chips, with a permeabilization time of 6 minutes identified as optimal. Subsequently, a single 10 µm frozen section was carefully attached to Stereo‐seq GE chips (0.5cm×0.5 cm), followed by fixation, fluorescent dye staining, permeabilization, and in situ reverse transcription. Amplification and library construction were performed on the obtained cDNA, followed by high‐throughput sequencing of the library on the DNBSEQ‐T7 platform.

### Stereo‐Seq Data Analysis and Visualization

Stereo‐seq data pre‐processing was performed based on established methodologies outlined in prior literature.^[^ ^15^ ^]^ and transcripts were merged according to bin50 for subsequent analysis and visualization. Cell clustering was conducted utilizing the DimPlot function in Seurat software, followed by the analysis and visualization of the expression levels of specific biomarkers at spatial positions using the SpatialDimPlot function.

### Lentivirus Interference of Organoids

The shRNA lentivirus targeting the SOX3 gene (target sequence: CGCAGGCAAGAGTAGTGCGAA) and the corresponding negative control lentivirus CON313 (target sequence: TTCTCCGAACGTGTCACGT) were generated by Genechem company based on the GV493 vector (component sequence: hU6‐MCS‐CBh‐gcGFP‐IRES‐puromycin). In the early stage of organoid induction, the organoids were transfected with lentivirus twice. Organoids were transfected with lentivirus twice during the early stages of induction. Specifically, on day 0, virus solutions were added to POCM1 at a multiplicity of infection (MOI) of 50 in the presence of HitransG A infection enhancer, and a similar procedure was repeated for POCM2 on day 3 at an MOI of 20.

### Quantification and Statistical Analysis

Statistical analyses were conducted utilizing GraphPad Prism 10.1 software, with data presented as means ± SD. The normality of all datasets was assessed using the Shapiro‐Wilk test and Kolmogorov‐Smirnov test, confirming that all data followed a normal distribution. Comparisons between the paired samples, two groups or multiple groups were conducted by the paired t‐test (two‐tailed), the student's t‐test (two‐tailed, if the variances were not equal, Welch's t‐test was used) or the one‐way ANOVA test with post hoc Bonferroni's method (If the variances were not equal, Brown‐Forsythe and Welch's ANOVA test with post hoc Dunnett's T3 multiple comparisons test was used), respectively. Two‐way ANOVA was applied to analyze the effects of two independent variables on the dependent variable, with post hoc Bonferroni's test to assess differences between multiple groups. For every bar graph, at least 3 independent experiments were conducted in distinct batches. Differences with *p* < 0.05 ^*^, *p* < 0.01 ^**^, and *p* < 0.001 ^***^were considered statistically significant.

### Ethics Approval Statement

The study has been approved by the ethics committee of Chinese PLA general hospital (Approval number: S2023‐290‐01).

## Conflict of Interest

The authors declare no conflict of interest.

## Author Contributions

S.W., D.J., and Y.X. contributed equally to this work. S.W.: methodology, investigation, visualization, writing‐original draft; D.J., Y.X., L.Y.: investigation, data curation, and formal analysis; Q.Q., H.Z.: software, validation; J.J.: conceptualization and project administration; X.J.: project administration, resources, writing‐review & editing; QG: conceptualization; funding acquisition; supervision; writing‐review & editing. All authors have reviewed and approved the final manuscript.

## Supporting information



Supporting Information

Supplemental Table 1

Supplemental Table 2

Supplemental Table 3

Supplemental Table 4

Supplemental Table 5

## Data Availability

Additional information necessary for the reanalysis of the data presented in this paper can be obtained from the corresponding author upon request.
