# Supplementary material for: Human Pituitary Organoids: Transcriptional Landscape Deciphered by scRNA‐Seq and Stereo‐Seq, with Insights into SOX3's Role in Pituitary Development
Source: Adv Sci (Weinh). 2025 Feb 14;12(14):2414230. doi: 10.1002/advs.202414230 (PMC11984888; doi:10.1002/advs.202414230)
Supplement: Supplementary file 1 — Supporting Information [file ADVS-12-2414230-s006.docx]

**
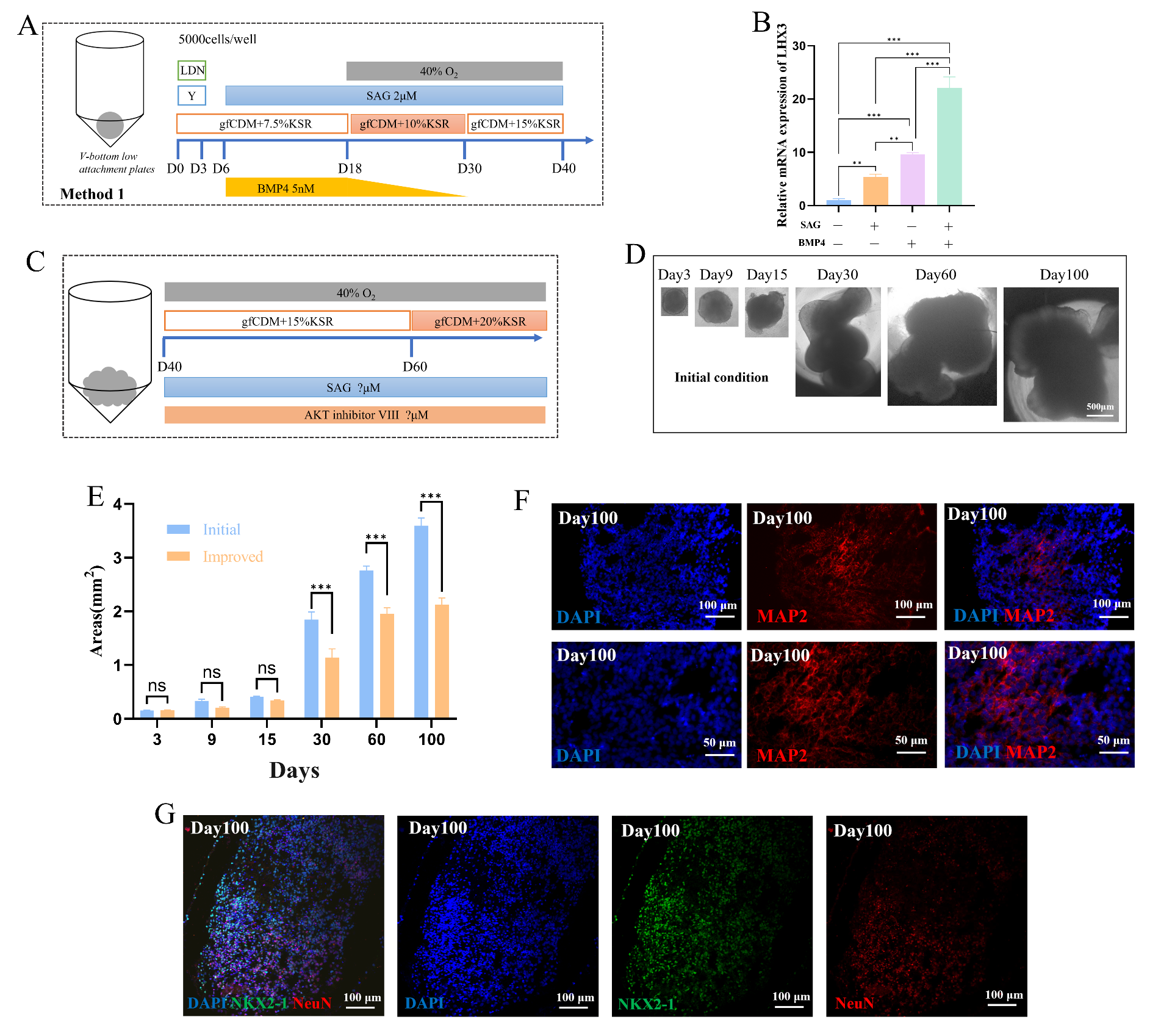
**

**Figure S1.** **Supplementary information on the initial culture protocol for three-dimensional pituitary organoids**

(A) Initial culture protocol for three-dimensional pituitary-placode organoids, named as the Method 1.

(B) Impact of adding 2 μM SAG (Day6-40) and/or 5 nM BMP4 (Day6-18) on the expression levels of LHX3 mRNA within organoids on day 40 (N = 3, mean ± SD, one-way ANOVA test with post hoc Bonferroni’s method).

(C) Schematic diagram of culture conditions for organoids after day 40.

(D) Bright-field images of the organoids cultured by the initial protocol (Scale bars: 500 μm).

(E) Comparison of the size between organoids obtained from the initial and improved protocols on days 3, 9, 15, 30, 60 and 100 (N = 5, mean ± SD, two-way ANOVA test with post hoc Bonferroni’s method).

(F) MAP2 Expression within organoids on day 100, revealed by immunofluorescence staining (Scale bars: 50μm, 100 μm).

(G) NKX2-1 and NeuN Expression within organoids on day 100, revealed by immunofluorescence staining (Scale bars: 100 μm).

**
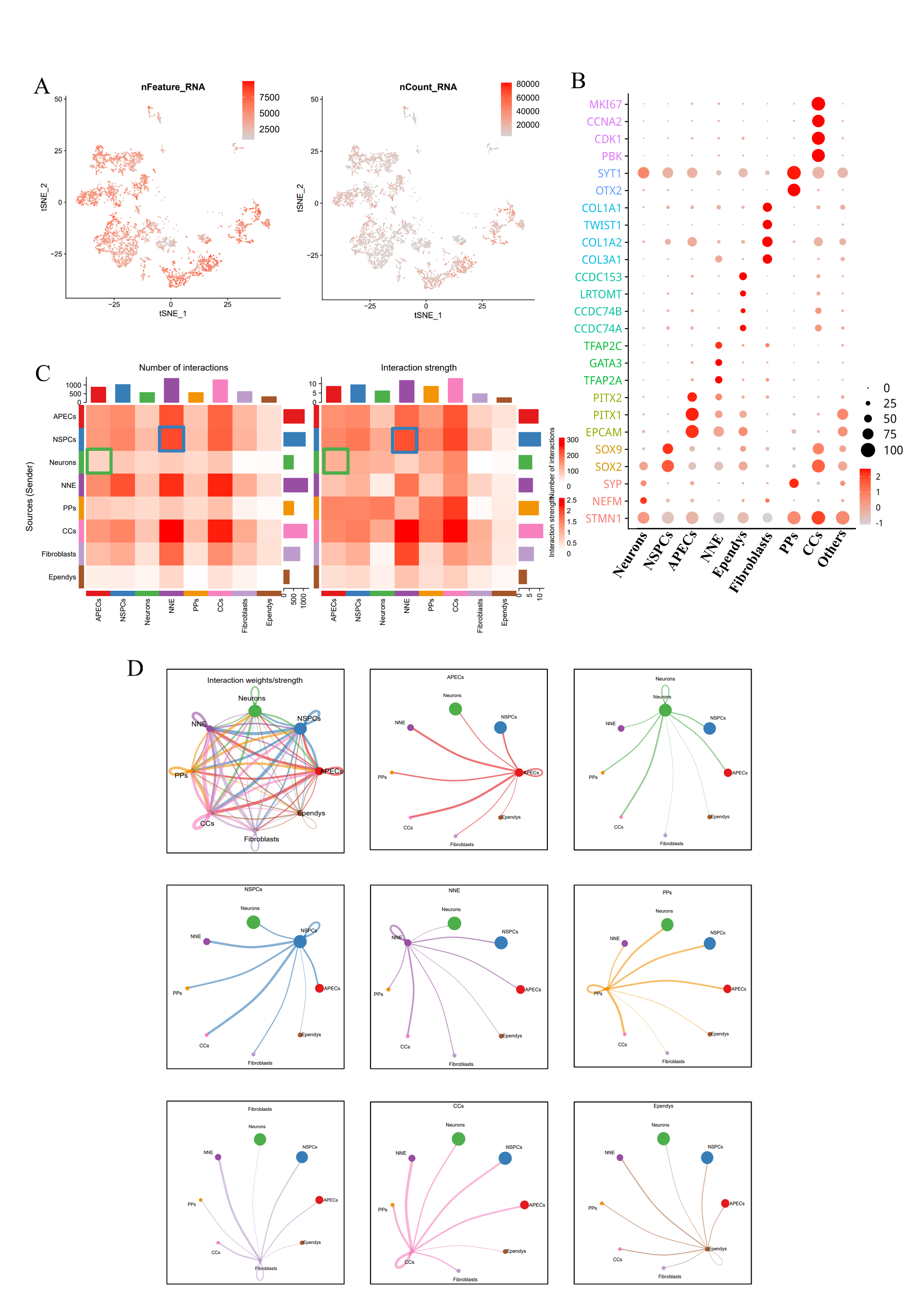
**

**Figure S2.** **Supplementary information on scRNA-seq analysis**

(A) The nFeature_RNA and nCount_RNA projection on the t-SNE plot. Deeper red, higher expression.

(B) Display of specific genes in nine clusters using the dot plot. Dot size, pct.exp; dot color, avg.exp.scaled.

(C) Global heatmap of the interactions between eight clusters, with the left image representing the number of interactions and the right image representing the interaction strength. The blue box represents the effect of NSPCs on NNE, and the green box represents the effect of Neurons on APECs.

(D) Local circle plot of the interactions between different clusters.

**
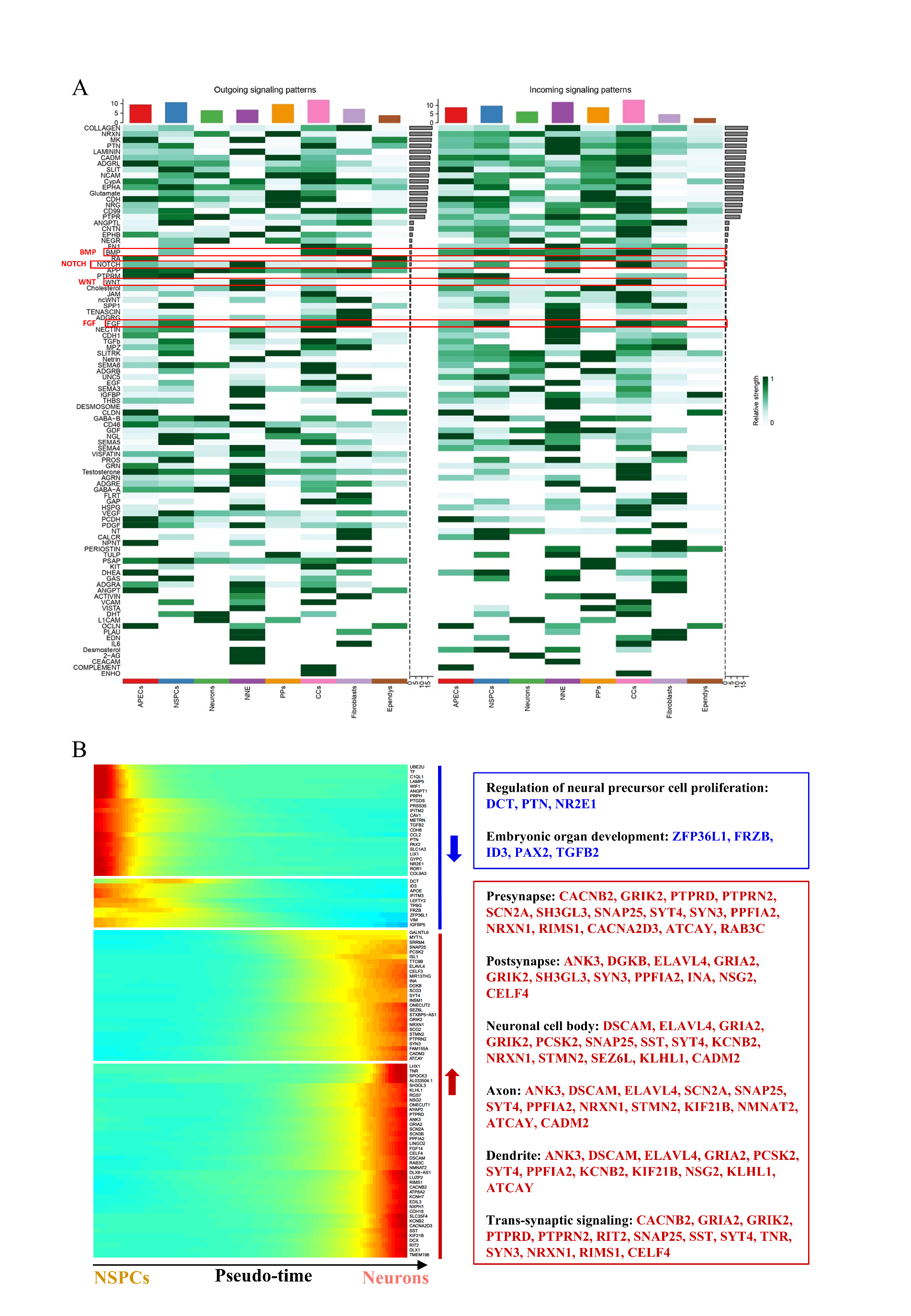
**

**Figure S3.** **Supplementary information on cell communication analysis and pseudo-time analysis**

(A) Pathway heatmap of the interactions between eight clusters, with each row representing a pathway and each column representing a cell type. The darker the color, the stronger the interaction. The left image shows the strength of the cluster where the ligand is located, while the right image represents the strength of the cluster where the receptor is located. The BMP4, NOTCH, WNT, and FGF pathways were outlined in red boxes.

(B) Heatmap displays the top 100 differentially expressed genes along the pseudo-time axis. Each row represents a gene, and the horizontal axis represents the pseudo-time axis. Blue represents genes with decreased expression along the pseudo-time axis, while red represents genes with increased expression along the pseudo-time axis.


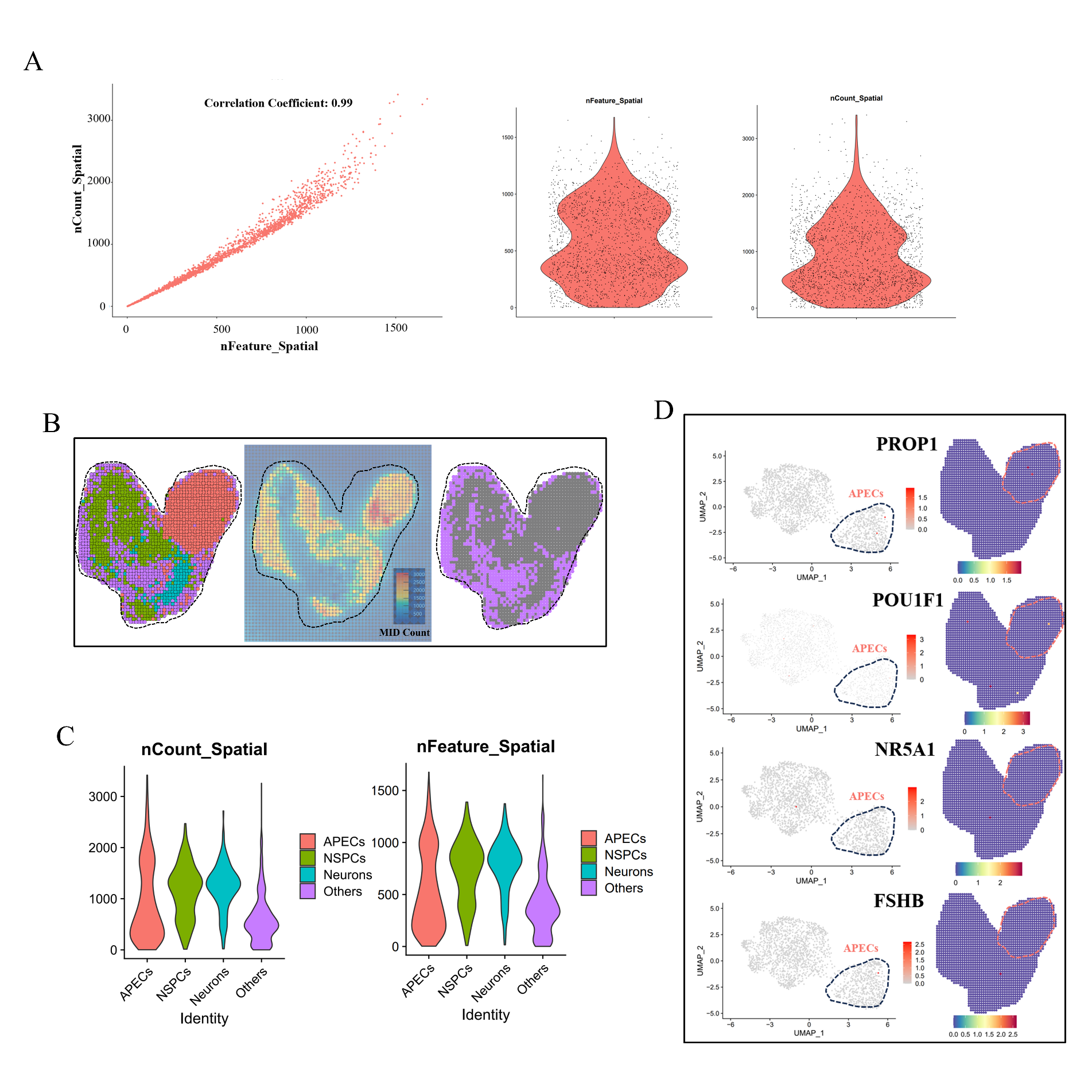


**Figure S4.** **Supplementary information on Stereo-seq analysis.**

1. nFeature, nCount and nFeature-nCount correlation of Stereo-seq data.

(B) Spatial distribution of the cluster “Others”. MID Count (Molecular Identifier Count): the gene expression level corresponding to the current coordinate.

(C) Violin diagrams showed nFeature and nCount of the four clusters.

(D) Expression of PROP1, POU1F1, NR5A1 and FSHB projected on the UMAP plot and their spatial distribution.

**
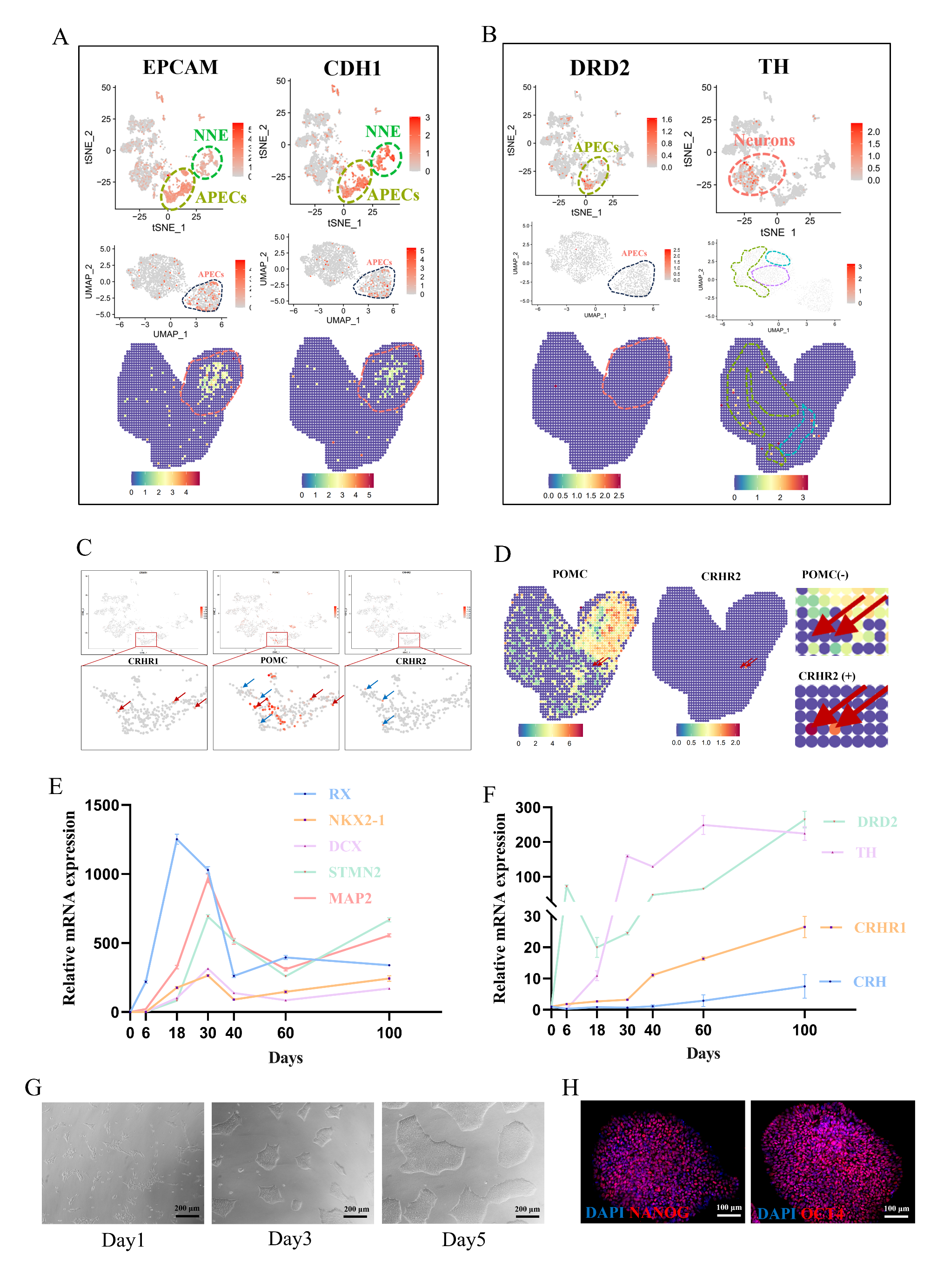
**

**Figure S5.** **Other supplementary information.**

(A) Expression of EPCAM and CDH1 projected on the t-SNE, UMAP and spatial plots. The t-SNE plot was generated from scRNA-seq, while the UMAP and spatial plots were generated from Stereo-seq.

(B) Expression of DRD2 and TH projected on the t-SNE, UMAP and spatial plots.

(C) Expression of CRHR1, POMC and CRHR2 projected on the t-SNE plot. Red arrow: CRHR1(+) POMC (+) dots; blue arrow: CRHR2(-) POMC (+) dots.

(D) Spatial distribution of POMC and CRHR2. Red arrow: CRHR2(-) POMC (+) dots.

(E-F) Relative mRNA expression levels of organoids across different days (N = 3, mean ± SD).

(G) Bright field images of human iPSCs on the 1st, 3rd, and 5th day after resuscitation (Scale bars: 200 μm).

(H) NANOG and OCT4 Expression in human iPSCs, revealed by immunofluorescence staining (Scale bars: 100 μm).
